# The Relationship between rs534654 Polymorphism in TMEM165 Gene and Increased Risk of Bipolar Disorder Type 1

**DOI:** 10.22088/IJMCM.BUMS.10.2.155

**Published:** 2021-09-01

**Authors:** Asmaolhosna Amini, Sara Sadat Aghabozorg Afjeh, Behzad Boshehri, Safar Hamednia, Parisa Mashayekhi, Mir Davood Omrani

**Affiliations:** 1 *Sara Medical Genetic Laboratory, Tehran, Iran.*; 2 *Department of Medical Genetics, Shahid Beheshti University of Medical Sciences, Tehran, Iran.*; 3 *Department of Forensic Medicine and Toxicology, Urmia University of Medical Sciences, Urmia, Iran. *; 4 *Department of Psychiatry, Urmia University of Medical Sciences, Urmia, Iran. *; 5 *Tajrish Research Center Pasteur Institute of Iran, Tehran, Iran. *; 6 *Urogenital Stem Cell Research Center, Shahid Beheshti University of Medical Sciences, Tehran, Iran.*

**Keywords:** Bipolar disorder, genetics polymorphism, TMEM165

## Abstract

Bipolar disorder (BD) is a major health care concern worldwide. There are some reports showing an association between genes and their variants involved in circadian rhythm; clock and clock related genes function and development of BD in patients. Therefore, the aim of this study was to investigate the possible association of rs534654 variant on* TMEM165* (transmembrane protein 165) gene with the risk of BD. Genotyping of the rs534654 was carried out using the tetra primers- amplification refractory mutation system-PCR (4P-ARMS-PCR) method in 203 patients with BD type 1 and their healthy and normal counterpart. The frequency of the G and A alleles of rs534654 polymorphism was 53% and 47%, respectively in patients. Genotype frequency in patients in comparison with control subjects was 5.4% vs 2.5% for the AA homozygous; 11.3% vs 80.8% for the GG homozygous; and 83.3% vs 16.7% for the heterozygous AG. Statistical analysis showed a significant diﬀerence in frequencies between the control and patient groups (P = 0.001). Based on this finding, it is possible to conclude that the impairment in the rs534654 single nucleotide polymorphism in *TMEM165* gene is associated with the risk of BD development.

Bipolar disorder (BD) is one of the most significant (prevalent) disabling mental diseases affecting 1–4% of the population worldwide (1-3). It is a complex disease associated with mood disorders (MD), depression, and schizophrenia (3). BD is determined by mood episodes and recurrences varying from depression to mania (4, 5). During the manic episodes, the need for sleep is reduced whereas depression episodes’ experience hypersomnia (6, 7). Genetics background is complicated in BDs with high heredity (H_85%), and it involves many genes and potential interactions between them and environmental factors (4). It has been shown in our previous reports that changes in BDNF (brain-derived neurotrophic factor), ADCY2 (adenylate cyclase 2) genes play an important role in the development of BD (8, 9). The human body is ruled by several sorts of rhythms. In addition to previously described genes and their controlling pathways, in mammals, the circadian rhythms are controlled by the brain (10). The central core of endogenous circadian clock is located in the superchiasmatic nuclei of the hypothalamus (11). Dysfunction of circadian rhythm, clock and clock related genes, all affect the pathophysiology of BD (12). Clock gene transcription and translocation, play an important role in circadian rhythm (13, 14).

The TMEM165 gene is one of the genes adjacent and overlapping with a clock gene which is located on chromosome 4 but transcribed from the opposite strand (15-17), and it is possibly involved in circadian cycle. A large number of classical genetic association studies revealed the role of circadian genes in predisposition to MD (18, 19). Human TMEM165 gene encodes 7 transmembrane protein domains which are calcium/proton transporter. Moreover, it has a role in regulating Ca^2+ ^and pH lysosomal homeostasis, and is mainly located in Golgi apparatus (16, 20, 21). It may play an indirect role in protein N-glycosylation (16). Recent studies have demonstrated that mutation in TMEM165 gene is associated with the rare autosomal recessive disorder “congenital disorders of glycosylation “(CDG) development in the affected people. Imperfection in TMEM165 contributes to defects in metabolic processes by impaired galactosylation and sialylation of total serum N-glycoprotein (22).

Single nucleotide polymorphisms in core circadian clock genes have been associated with psychiatric disorders (such as autism spectrum disorder, schizophrenia, anxiety disorders, major depressive disorder, BD, and attention deficit hyperactivity disorder) (23). This single nucleotide polymorphism rs534654 is located in TMEM165 which is a part of three-way interaction associated with BD (24). A previous study has assessed the association between rs534654 and disrupted sleep/wake cycles in BD patients (25). The authors reported that rs534654 might be related to weight loss in major MD patients but no significant association with anxiety/somatization symptoms such as block, sleep, maier, core and cognitive symptoms was observed.

The rs534654 polymorphism is an intron variant of TMEM165 gene with reference allele A, C, G and T. Based on the results of previous studies, the present study attempted to explore the relationship between the single nucleotide polymorphism (SNP) of *TMEM165* gene (rs534654) and elevated risk of BD.

## Materials and methods

**Study subjects**

In the present study, 203 individuals who were admitted to the Shahid Beheshti’s Imam hossein Teaching Hospital in Tehran-Iran by the psychiatrists and clinical staff in psychiatry wards were selected. All patients’ selection was based on the criteria mentioned in Manual of Mental Disorder; Fourth Edition-Text Revised (DSM IV-TR) and DSM-5 diagnosis of BD version (26). The same number of people without any history of mental illness was selected as healthy normal controls. The duration of the illness and controls’ age was 15-74 years (illness 43.23±11.2 years; control 36.63 ± 9.42 years). Patients with schizophrenia, autism spectrum disorder and intellectual disability were excluded. The patients had been controlled by lithium therapy at least 6 months, and they tipically consumed 600-900 mg/day dose. Their serum concentrations of lithium were assessed by urine screening (excretion from kidney). The typical effective serum concentrations were between 0.5-1.2 mEq/liter (27). The report on Helsinki of the World Medical Association have been followed in this study, and informed consents were signed by all participants. The Ethics Committee of Shahid Beheshti University of Medical Sciences (IR.SBMU.MSP.REC.1398.344).

**Genotyping methods**

Three milliliters of peripheral blood were obtained from all individuals in EDTA-contained tubes, and genomic DNA was extracted from each sample by means of salting out method. Genotyping of the rs534654 of the *TMEM165* gene was carried out using the tetra primers-amplification refractory mutation system-PCR (4P-ARMS-PCR) method in Applied Biosystems™ Veriti™ thermal cycler (Applied biosystems, USA). The sequences of inner and outer primers, their annealing temperature and the expected amplicon sizes for G and A alleles are shown in Table 1. A total volume reaction of 20 μL containing 50–100 ng of DNA template and 10 μL Taq DNA Polymerase 2 Master Mix Red (Ampliqon, Denmark) plus 5 pmol/L of outer primers, 10 pmol/L of inner primers were prepared. The PCR program started with the denaturing step (95°C for 5 min) followed by 35 cycles (95°C for 35 s, 62°C for 30 s, and 72°C for 30 s). Finally, reactions were incubated for 10 min in 72 °C as the final extension step. PCR products were interpreted by 2% agarose gel electrophoresis to visualize the specific bands associated with each genotype.

Amplification of rs534654 alleles generated 104, 160, and 210 bp bands for A allele, Gallele, and outer primers, respectively on gel electrophoresis. The PCR products were further approved by random sequencing of 10% of the obtained genotypes using the ABI 3730 DNA analyzer (Macrogen, Korea)

**Statistical analysis**

The Microsoft Excel 2019 and SPSS 24.0 statistical software (SPSS, Chicago, IL) were applied for statistical analysis of this case–control study. Both patients and control groups were analyzed using Chi-square (χ2) test to determine the fitness to the Hardy– Weinberg equilibrium. The χ2 test was also used for comparing genotype and allelic frequencies between the BD subjects and controls. The P-values were two-sided and a P < 0.05 was considered as statistically significant differences in all analyses.

## Results

The subjects in the control and patient groups were compared for demographic features such as sex and age. The mean age of the patients and controls were 43.23±11.2 years and 36.63 ± 9.42 years respectively. The demographic data of the selected patients showed that more than 53.2 percent of the cases were males.

**Evaluation of demographic indicators**

Differences between groups were assessed by t-test with Mann-Whitney formula (MS). There was no significant difference between the patient and control groups in terms of age index (P = 0.10). There was no significant difference between men and women (P = 0.94, 0.25).

**Table 1:** Sequences of primers used for genotyping

**Primer sequence **	**Tm**	**Annealing Temperature**	**Amplicon size (bp)**
Forward inner primer (G allele): 5′-GAATCAAGGATTTATCCAGTGAACACAGGC-3′	64°C	62°C	160
Reverse inner primer (A allele): 5′-CTGAGTGTCTGCTTTGCTCAGGGAA -3′	64°C	62°C	104
Forward outer primer 5′-GAATAGTATGCCTCTGCTTCCTGGGA-3′	64°C	62°C	210
Reverse outer primer 5′-GCATGTTCCTTCTCCCACAAAACAAAATC-3′	64°C	62°C	210

**Table 2:** Comparison of sociodemographic and clinical features of BD patients

**Features**	**Male (n=108)**	**Female (n=95)**
**Marital status**		
Single	42	33
Married	53	46
Divorced	7	10
NA	6	6
**Age at illness Onset (%)**		
Childhood (before 13)	2 (1.9)	1 (1)
Adolescence (13-18)	11 (10.2)	12 (12.6)
Young adulthood (19-29)	43 (39.8)	39 (41)
Old adulthood (after 30)	37 (34.2)	35 (37)
NA	15 (13.9)	8 (8.4)
**Family History Of Psychiatric Disorders (%)**	104 (51.2)	
NA	99 (48.8)	

**Fig. 1:**
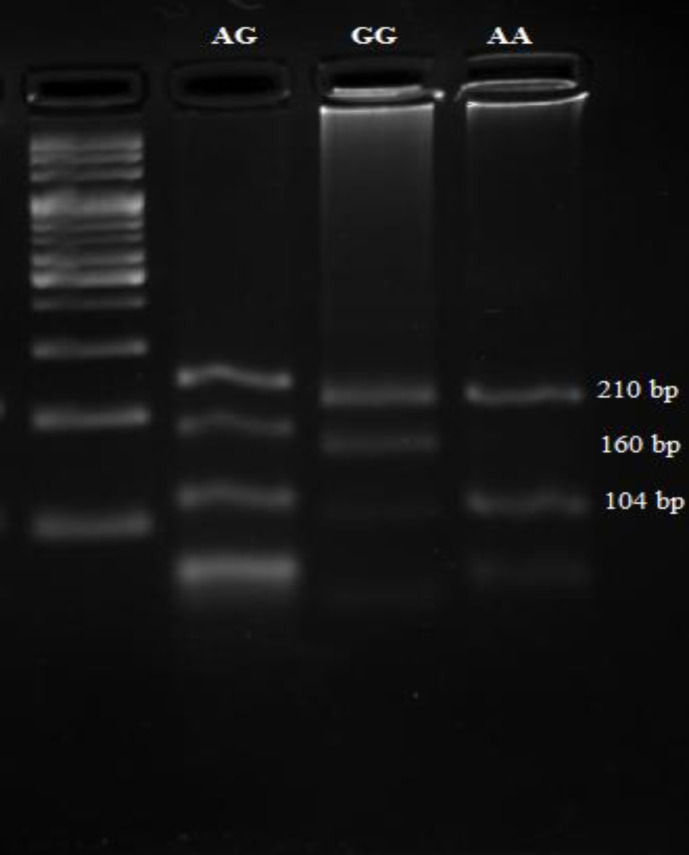
**Results of rs534654 genotyping by tetra-primer amplification-refractory mutation system -PCR methods. **Specific bands related to G and A alleles of the rs534654 SNP are shown

In addition, there was a significant difference between affected patients based on the social background such as being single, married or divorced, their education level, age at illness onset, family history of psychiatric disorder (Table 2) (28).

The specific band related to each genotype of the rs543654 SNP is shown in Figure 1.

**Table 3:** Genotypic model and allelic analysis of relationship between rs534654 polymorphism and bipolar disorder

		**Case** **n=203**	**Control** **n=203**	P-value (Pearson Chi-Square Test)
	**Genotype ** **(%)**			
**rs534654**	AA	11 (5.4)	5 (2.5)	.001
**rs534654**	AG	169 (83.3)	34 (16.7)	.001
**rs534654**	GG	23 (11.3)	164 (80.8)	.001
	**Allele (%)**			
**rs534654**	A	191 (47)	44 (11)	.001
**rs534654**	G	215 (53)	362 (89)	.001

The frequency of the G and A alleles of rs534654 polymorphism was 53% and 47%, respectively in the patients. Genotype frequency of the rs534654 polymorphism in patients in comparison with control subjects was 5.4% vs 2.5% for the AA homozygous; 11.3% vs 80.8% for the GG homozygous, and 83.3% vs 16.7% for the heterozygous AG. This statistical analysis shows a significant diﬀerence in frequencies between the control patient groups (P = 0.001). 

A positive association between SNP rs534654 and BD in the genotypic distributions (P = 0.001), (Table 3) was observed. 

## Discussion

BD has a complex genetic background. In the present study, it was found that more than 51.2% of patients had related history in their family, supporting the fact that a significant relationship may exist between heredity and BD (4, 29). It was reported that the largest onset age of BD is between 20-40 years old (30), which in the present study has also been the highest age of onset in patients group.

The present study assessed the association between the rs534654 in *TMEM165* and the risk of BD type I. Results showed a significant association between this SNP and BD development. SNP rs534654 in *TMEM165* gene is near the *CLOCK* gene which is a part of circadian genes. Several studies have demonstrated association between the role of circadian genes and the risk of MD. Indeed, the rs534654 polymorphism is part of a three-way interaction associated with BD (24). There is a multi-locus interaction between rs6442925 at the 5’ end of the upstream *BHLHB2* (basic helix-loop-helix domain containing, class B, 2) gene and rs1534891 at the *CSNK1E* gene. Similarly, rs534654 at the 3’ end of the clock gene (*TMEM165* gene) is significantly associated with BD in these studies (25). As mentioned before, the *TMEM165* gene is one of the genes located near the clock gene and possibly involved in the circadian cycle, and encodes a calcium / proton transporter protein that is involved in calcium homeostasis and lysosomal pH (20, 21). Calcium homeostasis is implicated in several physiologic processes, such as the homeostasis of the immune modulation as well as in several inflammatory processes (31). Mutations in *TMEM165* gene due to impairment of galactosylation and sialylation of N-glycoproteins are related to CDG (22). Most CDG are caused by defects in the glycosylation complex components, but the transgenic protein encoded by this gene is a Golgi protein that is involved in ionic homeostasis and vesicle transportation in the Golgi apparatus (32).

N-glycosylation is a post-translational change for many proteins and lipids with an oligosaccharide. This function regulates many biological processes, including intercellular communication, cell adhesion, protein folding, protein placement, and protein activity (33). Another important role of N-glycosylation is in the regulation of the immune system. The activity of one of the most important molecules in the humoral immune response ‘immunoglobulin IgG’ is strongly influenced by its N-glycan composition. It was demonstrated that the addition of galactose and sialic acid to IgG N-glycans encourages the anti-inflammatory cascade, while the presence of nuclear fucose regulates antibody-dependent cellular cytotoxicity, and completes the process (34).

Evidence suggests that dysfunction of immune system and inflammation are associated with depressive disorder. A meta-analysis study found that patients with depressive disorder had fewer immune cells than healthy people. Elevated pro-inflammatory cytokines, including interleukin-1, interleukin-6, and tumor necrosis factor, have been associated with depression (21,35) and have been shown to regulate the hypothalamic-pituitary-adrenal axis as well as neurotransmitter release (36). The potential role of N-glycosylation for other neurological disorders has also been suggested. In patients with schizophrenia, stimulators of glutamate transporters, stimulatory amino acids 1 and 2, are less glycosylated in the prefrontal cortex, and genes involved in N-glycan biosynthesis show altered expression (37). Different types of cells, including neurons, are coated with a complex structure of carbohydrates that facilitates their communication with other cells and the environment (38). On the other hand, gene expression studies show that *TMEM165* gene has significant expression in different parts of the brain with very high expression in the spinal cord of the brain (39). 

In the present study, the rs534654 variant of this gene, which has previously been shown to be associated with circadian rhythm disorders, was shown to be associated with an increased risk of BD.

Based on pivotal role of *TMEM165* in a calcium/proton transporter protein, importance of calcium ions in nerve conduction and the immune modulation, and the relation of immune system dysfunction with depressive disorder, as well as the relation between N-glycosylation and mental and neurological disorder, it is not unexpected that rs534654 variant of this gene is associated with risk of BD, and impairment in this gene may increase the risk of BD.

According to the results of this study, rs534654 variant of *TMEM165* could be considered as a potential risk factor of BD. Additional studies are also necessary to find other associated variants of *TMEM165* gene and understanding the underlying mechanism by which the rs534654 SNP influences the susceptibility to BD. 

## Conflict of Interest

Authors declare no conflict of interest.
